# Exploring the Drug Repurposing Versatility of Valproic Acid as a Multifunctional Regulator of Innate and Adaptive Immune Cells

**DOI:** 10.1155/2019/9678098

**Published:** 2019-03-14

**Authors:** Rodolfo Soria-Castro, Alejandro Schcolnik-Cabrera, Gloria Rodríguez-López, Marcia Campillo-Navarro, Nahum Puebla-Osorio, Sergio Estrada-Parra, Iris Estrada-García, Rommel Chacón-Salinas, Alma D. Chávez-Blanco

**Affiliations:** ^1^Departamento de Inmunología, Escuela Nacional de Ciencias Biológicas, Instituto Politécnico Nacional (ENCB-IPN), Mexico City, Mexico; ^2^Subdirección de Investigación Básica, Instituto Nacional de Cancerología (INCan), México City, Mexico; ^3^Laboratorio de Inmunología Integrativa, Instituto Nacional de Enfermedades Respiratorias Ismael Cosio Villegas, Mexico City, Mexico; ^4^Department of Lymphoma and Myeloma, The University of Texas, MD Anderson, Cancer Center, Houston, TX, USA; ^5^Unidad de Desarrollo e Investigación en Bioprocesos (UDIBI), Escuela Nacional de Ciencias Biológicas, Instituto Politécnico Nacional (ENCB-IPN), Mexico City, Mexico

## Abstract

Valproic acid (VPA) is widely recognized for its use in the control of epilepsy and other neurological disorders in the past 50 years. Recent evidence has shown the potential of VPA in the control of certain cancers, owed in part to its role in modulating epigenetic changes through the inhibition of histone deacetylases, affecting the expression of genes involved in the cell cycle, differentiation, and apoptosis. The direct impact of VPA in cells of the immune system has only been explored recently. In this review, we discuss the effects of VPA in the suppression of some activation mechanisms in several immune cells that lead to an anti-inflammatory response. As expected, immune cells are not exempt from the effect of VPA, as it also affects the expression of genes of the cell cycle and apoptosis through epigenetic modifications. In addition to inhibiting histone deacetylases, VPA promotes RNA interference, activates histone methyltransferases, or represses the activation of transcription factors. However, during the infectious process, the effectiveness of VPA is subject to the biological nature of the pathogen and the associated immune response; this is because VPA can promote the control or the progression of the infection. Due to its various effects, VPA is a promising alternative for the control of autoimmune diseases and hypersensitivity and needs to be further explored.

## 1. Introduction

The short-chain 2-*n*-propyl-pentanoic fatty acid, also known as valproic acid (VPA), is soluble in organic solvents and stable at room temperature. VPA was created as an analogue of valeric acid, or pentanoic acid, extracted from *Valeriana officinalis* [1]. VPA is the most used drug for the multiple types of epilepsy, including tonic-clonic or grand mal seizures, complex partial seizures, tonic seizures including Lennox-Gastaut syndrome, and absence or petit mal seizures [2, 3]. Furthermore, this compound is used to treat manic syndrome and migraines [4], and due to its effect as a histone deacetylase inhibitor (HDACI), several studies have analyzed its potential therapeutic use for diseases such as HIV and cancer [3, 5, 6]. Although VPA might induce hepatotoxicity and teratogenicity, it is one of the safest anticonvulsant compounds in current use [7].

## 2. Generalities of Valproic Acid

### 2.1. Pharmacokinetics and Pharmacodynamics of Valproic Acid

VPA is a weak acid (pKa 4.95), and after oral or parenteral administration, it is absorbed almost completely, presenting a bioavailability of ≥80% [2]. Just as with endogenous free fatty acids, VPA is a molecule highly bound to proteins (87-95%), mostly to albumin, which results in a low clearance rate (6-20 mL/h/kg) [8]. However, its binding to plasmatic proteins diminishes with continuous administration, resulting in a free fraction of the drug, which is the only form that crosses the cellular membrane [2]. The peak of plasma VPA is achieved 4 hours post administration, with a half-life of 11-20 hours, depending on the clinical formulation [9]. After continuous oral treatment, patients usually present VPA plasma concentrations within a range of 40-100 *μ*g/mL (280-700 *μ*mol/L) [2].

VPA undergoes diverse biotransformation, which is evident by the presence of <3% of unchanged VPA in urine [2]. Conventionally, in humans, VPA elimination occurs through three major metabolic pathways: glucuronidation and mitochondrial *β*-oxidation account for 50 and 40% of the metabolism of VPA, respectively, while cytochrome P450- (CYP450)- mediated oxidation is only a minor route [8].

Due to its fatty acid nature, VPA is metabolized in the mitochondria. The primary metabolite of VPA, VPA-glucuronic acid (~30-50%), is generated by multiple UDP-glucuronyl transferases in the cytosol of hepatocytes [8, 10] and is excreted in the urine. VPA bioactivation requires the entry of the metabolite 4-ene-VPA into the mitochondria, with the formation of the 4-ene-VPA-CoA ester, and *β*-oxidation to form 2,4-diene-VPA-CoA ester [8]. Interestingly, the cytotoxic metabolite 2,4-diene-VPA-S-CoA couples with glutathione to form thiol conjugates, which deplete mitochondrial glutathione pools, joining afterward with CoA, leading to the inhibition of enzymes of the *β*-oxidation pathway [8]. However, while certain VPA metabolites are hepatotoxic, both 2-propyl-2-pentanoic acid and 2-propyl-4-pentenoic acid exhibit anticonvulsive effects almost as potent as those of VPA [8]. Finally, regarding CYP450 biotransformation, it is recognized that this compound is metabolized into two hydroxylated metabolites [11].

The antiepileptic effects of VPA mainly increase the availability of *γ*-aminobutyric acid (GABA) in the neuronal space. VPA raises GABA levels through an increase in glutamic acid decarboxylase (GAD), which synthesizes GABA through the inhibition of both the succinic semialdehyde dehydrogenase (SSAD) and the GABA transaminase (GABA-T) [2]. In addition, VPA reduces the release of the epileptogenic *γ*-hydroxybutyric acid [2]. On the other hand, VPA functions as an antiepileptic drug through the blockade of voltage-gated sodium, potassium, and calcium channels, thereby diminishing the continuous activation of neurons [8].

### 2.2. Neurological Effects of Valproic Acid

VPA was the third antiepileptic drug approved by the Food and Drug Administration, and unlike most of the antiepileptic compounds, it lacks nitrogen and a ring moiety [12]. Although VPA can be employed in either generalized or partial epilepsies, it is more effective in the former, mainly to treat absence seizures [13]. One of the primary mechanisms of action that VPA employs to modulate neuronal discharges is through the increase of GABA levels.

GABA is an inhibitory neurotransmitter in mammals that is released by the presynaptic cell and is recognized by GABA receptors located at the postsynaptic cell. The joining of GABA to its receptor results in chloride ion (Cl^−^) influx to the postsynaptic neuron, thus inhibiting the propagation of the nervous impulse due to hyperpolarization of the neuron [14]. This amino acid is synthesized in the brain by the metabolism of *α*-ketoglutarate to glutamate, which is in turn transformed into GABA by the GAD enzyme. VPA upregulates GAD and inhibits both SSAD and GABA-T, which degrade GABA. Additionally, VPA increases the expression of GABA receptors, thus promoting the extension of its neuronal effects [1].

A previous work in nerve fibers from *Xenopus laevis* exposed to low concentrations of VPA demonstrated a direct effect on reducing the conductance of both sodium and potassium at the central level, which led to a decrease in neuronal excitability [15]; this implies that VPA may act on several ion channels at the central level, which together can potentiate the hyperpolarization of the neuronal membrane.

### 2.3. Epigenetic Effects of Valproic Acid

Histones were considered structural elements for the formation of nucleosomes, without any other role. However, they are now recognized as important elements in epigenetic regulation, through covalent modifications in their amino terminal tails, which are exposed on the surface of the nucleosomes, allowing them to interact with nuclear factors [16–18]. This phenomenon, known as histone code, involves the combination of modifications in one or more histones to allow or impede the access to transcription factors and regulatory proteins, which modifies the expression pattern for genetic activation or silencing of genes, without changing the genotype [18].

Histone modifications include, among others, acetylation and methylation of lysine and arginine; phosphorylation of serine and threonine; ubiquitination and sumoylation of lysine; ADP ribosylation of glutamic acid; deamination of arginine; and isomerization of proline [19–21]. Histone acetyltransferases (HATs) and histone deacetylases (HDACs) are involved in the acetylation and deacetylation of lysine residues, modifying the charge in histone tails and promoting chromatin decondensation (acetylation) or packaging (deacetylation) [22, 23]. Such changes regulate DNA replication, transcription, and repair.

VPA induces the epigenetic inhibition of HDACs categorized as class Ia (HDAC1 and HDAC2), class Ib (HDAC3), class Ic (HDAC8), and class IIa (HDAC4, HDAC5, and HDAC7), leading to an increase in the acetylation of histones H2, H3, and H4, which modify the expression of genes associated with apoptosis, cell cycle, cell differentiation, and defense against tumor cells [8, 24, 25]. VPA displays cell-specific selectivity; for example, it attenuates the activity of HDAC6 and HDAC 8 in a model of cardiac hypertrophy [26], inhibits HDAC4/5 in a model of renal fibrosis [27], inhibits HDAC1/2 in stellate cells during chronic administration in a model of hepatic fibrosis [28], and inhibits HDAC3/4 in a model of penile fibrosis [29]. HDAC inhibition is associated with good prognosis for several neuronal pathologies because class I and II HDACs strongly impact neuronal function [3].

Furthermore, VPA can alter DNA methylation, carried out by DNA methyltransferases (DNMTs), which add a methyl group from S-adenosyl-L-methionine to the fifth carbon of cytosine of CpG dinucleotides, leading to transcriptional silencing [30]. Conversely, the ten-eleven translocation (TET) enzymes oxidize 5-methylcytosine to 5-hydroxymethylcytosine, promoting the reversal of DNA methylation and gene silencing [31]. VPA also decreases methylated DNA in different cell lines, including neuroblastoma, HEK 293, and HeLa [32–34]. In addition, VPA-treated mice showed increased DNA demethylation in their brains [35]. Recent evidence indicates that VPA favors the accumulation of 5-hydroxymethylcytosine in a model of neuronal differentiation, suggesting an increase in TET activity [36].

Currently, VPA and more HDACIs are therapeutic alternatives, and most of them have been approved for clinical use [37]. VPA is now accepted as a first-line treatment for epilepsy, bipolar disorder, and migraines; it is also undergoing a phase II clinical trial for the treatment of both leukemia and solid tumors [24, 38].

### 2.4. Metabolic Effects of Valproic Acid

VPA use relates to the dysregulation of the usual pattern of metabolite usage, particularly in the brain. Certainly, several studies have demonstrated that either chronic use or a single dose of VPA is enough to modify the consumption of glucose, fatty acids, and glutamine by brain cells.

The first and most evident metabolic effect of VPA is related to the chemical structure of this drug. Indeed, VPA is structurally similar to endogenous fatty acids, which makes it susceptible to mitochondrial fatty acid *β*-oxidation. Since 40% of VPA is biotransformed through *β*-oxidation, patients under chronic VPA treatment usually develop liver microvesicular steatosis [2]. This effect can be exacerbated in patients with congenital mitochondrial disorders [39].

VPA can alter the pattern of glucose consumption by brain cells, as observed in chronically treated patients with VPA, who showed a minimized glucose metabolism in their brains; this demonstrates a strong correlation between higher VPA concentrations and a reduction in the total glucose metabolism of the brain [40]. Rakitin et al. observed that patients receiving a single dose of intravenous VPA developed a significant decrease in serum glucose, without modification of the insulin levels [41].

Experiments performed in rats also showed that increasing doses of VPA diminished the glutamine availability in astrocytes [42]. Although this can be related to the rise in GABA concentration as a direct effect of VPA for the treatment of seizures, the reduction in glutamine levels limits the use of this amino acid as a substrate for the Krebs cycle, via *α*-ketoglutarate, to support oxidative phosphorylation under stress conditions [43]. Doses of VPA as low as 240 *μ*M block the oxidative phosphorylation in the rat liver mitochondria, having glutamate as a substrate [44].

Although little is known about the effect of VPA in the metabolism of immune cells, it is expected that a systemic treatment with VPA will have an effect on them. This is because, under an immunological response, immune cells shift their metabolite source to maximize energy efficiency while increasing both cell proliferation and specificity against harmful agents [45].

### 2.5. Other Therapeutic Effects of Valproic Acid

VPA exerts its functions through several molecular targets, supporting its potential employment for a wide range of pathologies other than epilepsy; those include psychological disorders such as depression, dependence on psychotropic substances, and manic behaviors; cancer and other chronic diseases; and immune disorders. Commonly, VPA is used alone, but in some instances, it is also employed in combination with other drugs. The majority of the more than 370 clinical trials using VPA are now in phase I or II, some of which have shown auspicious effects (Table 1).

A significant effect of VPA, along with some HDACIs, is their ability to induce apoptosis in tumor cells, both *in vitro* and *in vivo*, along with cell cycle and differentiation arrest, senescence, inhibition of angiogenesis [5], and an increase in the levels of stress molecules recognized by NKG2D [46]. Altogether, these modifications make tumor cells more susceptible to immune recognition.

According to recent findings, VPA can modulate different cellular and molecular pathways in immune cells, representing a new therapeutic potential for the treatment of diseases in which the immune response is crucial. We present a summary of some effects of VPA in immune cells in Table 2.

## 3. Effects of Valproic Acid on Cells of the Innate Immune Response

### 3.1. Monocytes

Some reports suggest that VPA promotes the differentiation of myeloid hematopoietic progenitors into the monocytic lineage. In CD34^+^ hematopoietic stem/progenitor cells (HSPCs), including erythroblasts, megakaryoblasts, and promyeloblasts, VPA increases CD11b and CD14 expression. VPA also decreases the expression of erythroid lineage markers, due to the increased levels in the transcription factor PU.1 and the reduction of transcription factors GATA-1, FOG-1, and SP1 [47]. In myelomonocytic cells of the U937 cell line, VPA induces differentiation into monocytes through an increase in H3 and H4 acetylation, as well as by inducing the expression of p21, which is required for monocyte development; this is correlated with an upregulation of the myeloid markers CD11a, CD11b, CD11c, CD13, CD18, and CD64 [48].

The activation of postdifferentiated monocytes in response to some stimuli tends to be affected by VPA. Human THP-1 monocytes treated with VPA and stimulated with LPS showed a reduction in the secretion of both IL-6 and TNF-*α* due to inhibition of NF-*κ*B activation [49]. This effect is similar in murine splenocytes, in which VPA reduces TNF-*α* concentrations in response to LPS [50]. Another study reported that VPA increased the proliferation and the expression of both CD11b and IL-18 in the U937 monocyte cell line [51].

In a U1 monocyte cell line infected with HIV-1 in the latency stage, Matalon et al. observed that VPA diminished the expression of the viral protein p24. However, in monocytes obtained from the peripheral blood of uninfected subjects, but stimulated with LPS, there was a reduction in the expression of the HIV coreceptor CCR5 at both mRNA and protein levels; this suggests a possible therapeutic effect driven by VPA in HIV infection [52]. However, other studies have suggested a different outcome, as VPA increased HIV replication in U937, U1 cells, and monocytes from the peripheral blood [53, 54].

### 3.2. Macrophages

VPA on its own can reduce the activity of HDACs 1, 2, and 3; the activation of NF-*κ*B; and p38 activation, which impair TNF-*α* and CCL-2 production by RAW264.7 murine macrophage cell line [55]. In addition, VPA blocked macrophage migration and downregulated macrophage responses against microbial stimuli through the inhibition of the proinflammatory cytokines TNF-*α*, IL-1*β*, IL-6, IL-18, IL-12, and IFN-*γ*; the PAMP receptors of the TLR, NOD, RIG-1, and C-type lectin families; the adapter molecules MyD88; the kinases IRAKs, MAP3Ks, and TRAF-1; phosphatases; and transcriptional modulators, as well as blocked macrophage migration [56].

**Figure 1:**
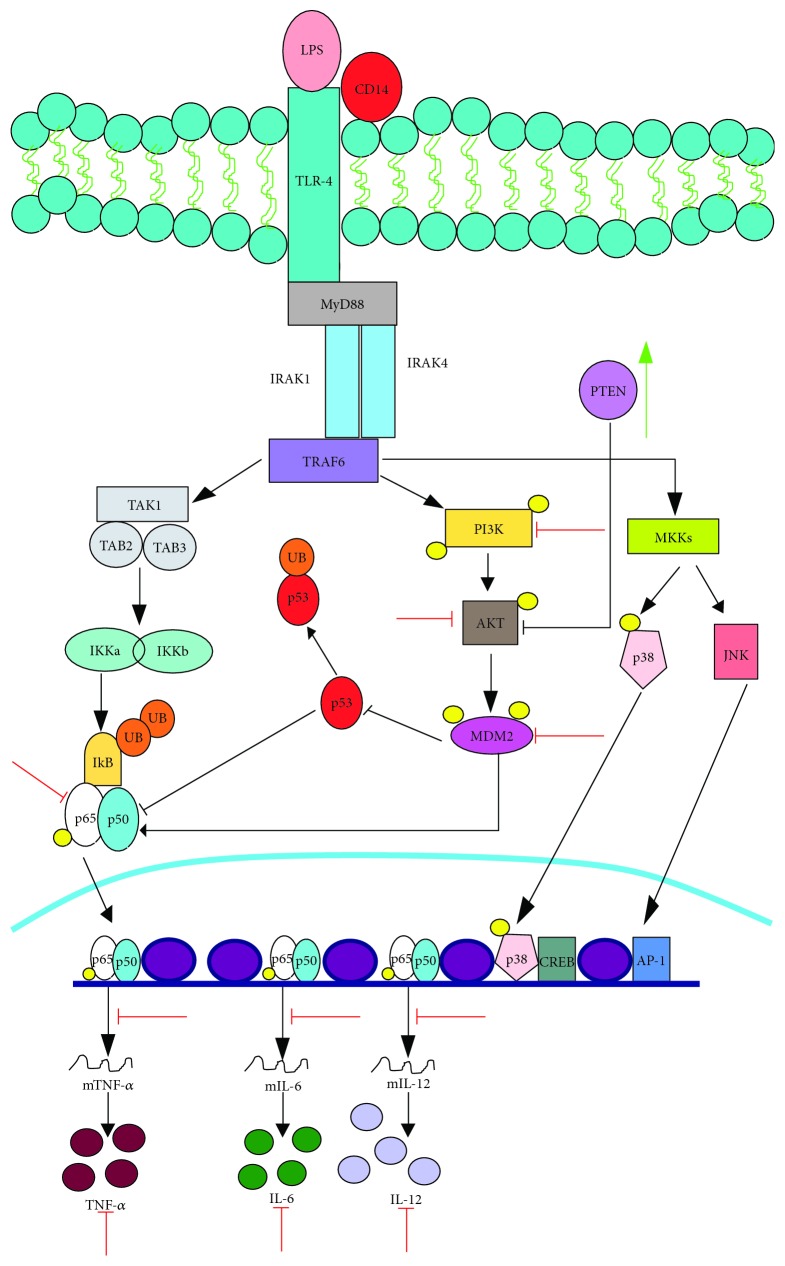
VPA inhibits LPS signaling in macrophages. VPA inhibits the production of proinflammatory cytokines (IL-6, IL-12, and TNF-*α*) after stimulation with LPS by affecting the phosphorylation of NF-*κ*B, PI_3_K, Akt, and MDM2. Therefore, there is an increase in the levels of the NF-*κ*B inhibitor p53 and in the levels of the negative regulator of Akt, PTEN. Green arrows indicate the processes, molecules, or mediators in the signaling pathway that are augmented and/or promoted by VPA. Red arrows indicate processes, molecules, or mediators in the signaling pathway that are inhibited by VPA.

VPA diminished the expression of nitric oxide (NO) in IFN-*γ*-treated RAW264.7 macrophages, due to a reduction in the inducible nitric oxide synthase (iNOS) enzyme. This effect is associated with the induction of VPA-driven acetylation on the transcriptional factor STAT1, which impedes its association to the promoter region of the iNOS gene [57]. Similarly, VPA reduces the production NO and iNOS production, as well as IL-6 and IL-12, in response to LPS (Figure 1) [58].

In bone marrow-derived macrophages (BMDMs) from BALB/c mice stimulated with the ligand of the TLR1/TLR2 heterodimer, Pam_3_CSK_4_, VPA reduced the production of TNF-*α*, IL-6, and IL-12p40 [59].

LPS activates the PI_3_K/Akt/MDM2 signaling pathway in macrophages, increasing the transcriptional activity of NF-*κ*B-mediated proinflammatory cytokines. However, VPA inhibits this pathway by reducing PI_3_K, Akt, and MDM2 phosphorylation. Because MDM2 ubiquitinates p53 for its degradation, increasing levels of p53 prevent NF-*κ*B activation, drastically reducing the levels of IL-6 and TNF-*α*; the induction of PTEN, a negative regulator of the PI_3_K/Akt pathway, potentiates this effect. Interestingly, VPA does not reduce TLR-4 or MyD88 expression or their physical interaction with PI_3_K (Figure 1) [60].

**Figure 2:**
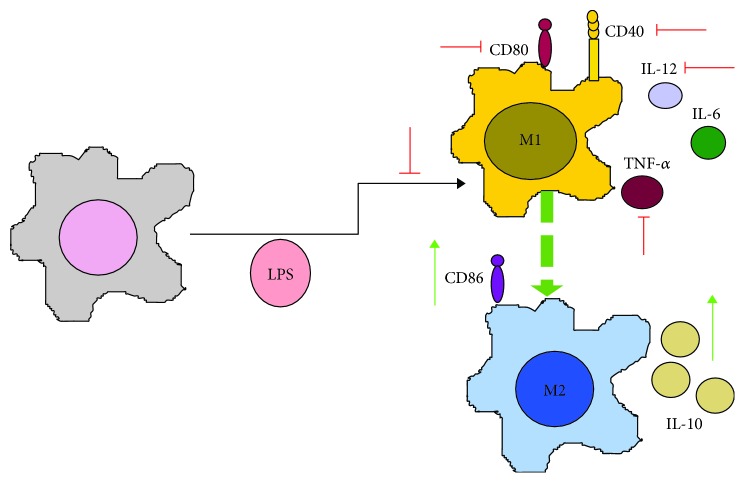
VPA induces M1-M2 polarization of macrophages in response to LPS. VPA polarizes macrophages from the M1 to the M2 immune phenotype in response to the stimulation with LPS by changing the cytokine profile (IL-12, IL-6, and TNF-*α* are reduced, while IL-10 increases), as well as the pattern of costimulatory molecules (from CD80 to CD86, and inhibiting CD40 expression). Green arrows indicate the processes, molecules, or mediators in the signaling pathway that are augmented and/or promoted by VPA. Red arrows indicate processes, molecules, or mediators in the signaling pathway that are inhibited by VPA.

Wu et al. employed BMDM and RAW264.7 macrophages and observed that VPA altered their response to LPS (Figure 2) by reducing IL-12p70 and TNF-*α* release, and by increasing IL-10 production and the costimulatory molecules CD40, CD80, and CD86. Therefore, VPA polarizes macrophages from a proinflammatory M1 to an anti-inflammatory M2 phenotype, which is unable to induce naïve TCD4^+^ differentiation into a Th1 profile, favoring a Th2 phenotype [61].

In contrast, VPA can exacerbate the RAW264.7 macrophage response to LPS by increasing the release of the nuclear factor HMGB1, which associates with the promotion of inflammatory processes. This effect is mediated by an increase in the expression of the GABA receptor, leading to phosphorylation of the transcription factor ERK1/2, without promoting either p38 or c-JUN phosphorylation [62].

Employing *in vitro* models of infection with extracellular bacteria, VPA reduces both the phagocytosis and elimination of *Escherichia coli* and *Staphylococcus aureus* by BMDM through the reduction in the expression levels of scavenger receptors, CD14, Dectin 1, CR4, TLR1, TLR2, TLR3, TLR4, TLR6, TLR8, and TLR9, and by inhibiting the generation of reactive oxygen species (ROS) due to the blockade of the expression of both NADPH oxidase and iNOS [63].

**Figure 3:**
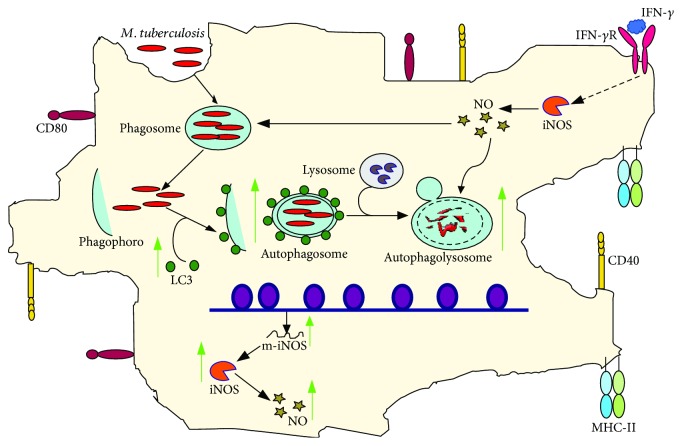
VPA promotes the control of *M. tuberculosis* infection in macrophages. VPA reduces *M. tuberculosis* load in infected macrophages through the induction of autophagy and by increasing NO induced by IFN-*γ*. Green arrows indicate the processes, molecules, or mediators in the signaling pathway that are augmented and/or promoted by VPA. Red arrows indicate processes, molecules, or mediators in the signaling pathway that are inhibited by VPA.

Contrary to established knowledge in extracellular bacteria, it appears that VPA promotes the control of infection by intracellular bacteria (Figure 3). Nieto-Patlán et al. demonstrated that the employment of VPA induced a reduction in *Mycobacterium tuberculosis* load in IFN-*γ*-activated J774A.1 macrophages, which correlated with the rise in NO production due to an increase in the expression of iNOS (Figure 3) [64]. In line with this, VPA promotes the intracellular elimination through autophagy of *M. bovis* BCG or *M. tuberculosis*, in infected RAW264.7 macrophages and human alveolar macrophages, respectively (Figure 3) [65]. The effect of VPA on autophagy induction was also found in human monocyte-derived macrophages and in murine alveolar macrophages infected with *M. tuberculosis* [66].

Interestingly, VPA reduced H37Rv *M. tuberculosis* load in THP-1 macrophages, and when this drug was combined with the first-line antituberculosis drugs rifampicin and isoniazid, it drastically reduced in the bacterial load [67]. Thus, VPA has a new therapeutic potential for the treatment of tuberculosis, as demonstrated by *in vitro* models of mycobacterial infection.

Apoptosis is one of the mechanisms that contribute to the resolution of acute inflammatory processes. In 2013, Montero et al. observed that peritoneal macrophages activated with LPS in the presence of VPA induced macrophage release of annexin A1 without inducing macrophage apoptosis. Remarkably, neutrophils suffered apoptosis in an annexin A1-dependent manner. On the other hand, VPA increased zymosan phagocytosis by macrophages and lowered the production of IL-6 and TNF-*α* in response to LPS [68].

A similar effect was observed by Tsolmongyn et al. in RAW264.7 macrophages, where VPA diminished cell viability and proliferation due to apoptosis induction and an increase in the expression of caspase 2, PARP3, Bax, and Bcl-2, as well as phosphorylated p53 [69, 70].

VPA is also able to modify the response of alternatively activated macrophage (AAM), a subclass of macrophage induced by IL-4 or IL-13 and whose importance lies during tissue repair and regulation of inflammatory processes. VPA-treated AAMs reduce their arginase activity and decrease the production of NO and IL-10 in response to LPS [71].

*In vivo* studies have demonstrated that VPA reduces macrophage infiltration in various models of inflammation. A rat model of spinal cord injury showed a diminished macrophage infiltration due to a reduced inflammation, which provided neuroprotection [72, 73]; likewise, VPA also reduced the macrophage infiltration in a mouse model of nephropathy [74]. In addition, a rat model of lung injury demonstrated that VPA impaired M1 macrophages and the expression of the antigen of nuclear proliferation and of oxidative stress markers, while favoring the proliferation of M2 macrophages; this was related to reduced inflammation and increased tissue repair [75].

According to some studies, macrophages from systemic lupus erythematosus (SLE) are incapable of removing apoptotic bodies, which results in enhanced inflammation and induction to M1 macrophages. In SLE patients, macrophages stimulated with apoptotic bodies and VPA showed a polarization toward an M2 phenotype, with a concomitant decrease in M1 macrophages. This was accompanied by an increase in both IL-10 and TGF-*β* and reduced levels of IL-1*β* and TNF-*α*. These phenotypic changes could be an adjuvant for the treatment of autoimmune diseases [76].

### 3.3. Dendritic Cells

Contrasting with the effect of VPA on myeloid progenitors for monocyte differentiation, early studies suggested that VPA disturbs the differentiation and maturation of dendritic cells (DCs) [77]. In C57BL/6 mice, VPA hinders the differentiation of bone marrow multipotent progenitors into plasmacytoid DCs (pDCs) and myeloid DCs; this is secondary to the reduced expression in the transcription factors PU.1 and IRF8 [78]. In addition, VPA reduces the expression of both CD86 and MHC-II [79].

**Figure 4:**
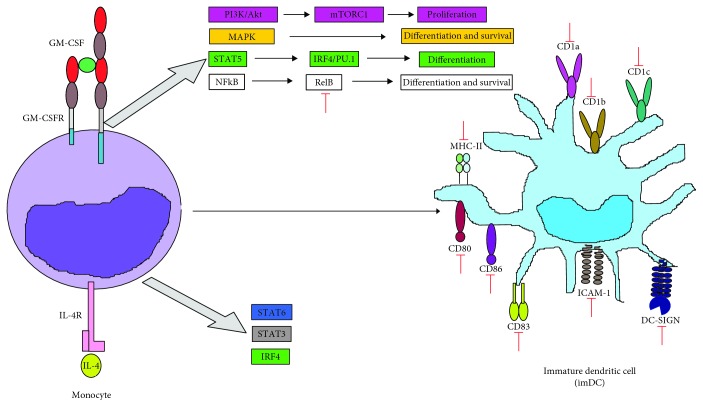
VPA inhibits monocyte to DC cell differentiation. VPA suppresses monocyte to DC differentiation after being stimulated with IL-4 and GM-CSF, by affecting RelB translocation (a noncanonic NF-*κ*B subunit) into the nucleus, which contributes to the reduction in the expression of diverse characteristic surface molecules from immature DCs, including costimulation (CD80, CD83, and CD86), adhesion (ICAM-1/CD54 and DC-SIGN), MHC-II (HLA-DR), and CD1 isoform (a, b, and c) molecules. Green arrows indicate the processes, molecules, or mediators in the signaling pathway that are augmented and/or promoted by VPA. Red arrows indicate processes, molecules, or mediators in the signaling pathway that are inhibited by VPA.

Nencioni et al. showed that VPA inhibited the differentiation of human monocytes into DCs (moDCs) via reduction of CD1a, CD80, CD83, CD86, ICAM-1, and DC-SIGN, correlating with a decrease in NF-*κ*B activity [80]. Similarly, Leu et al. showed that VPA decreased the expression of CD1a, CD1b, CD1c, CD83, and CD86 in moDCs (Figure 4) [81].

VPA can suppress moDC maturation in response to different stimuli. In this sense, VPA inhibits TLR-3 ligand activation, induced by poly I:C, as reflected by the decreased expression of CD1a, CD83, CD80, CD40, and DC-SIGN. This correlates with lower nuclear levels of NF-*κ*B, IRF-3, and IRF-8. This effect promoted less chemotaxis in response to CCL19, as well as a lower capacity to induce proliferation of allogenic peripheral mononuclear cells [80].

In LPS-activated moDCs, VPA induced a substantial reduction in the costimulatory molecules CD40, CD80, and CD86; in the chemokine receptor CCR7; and in the production of both cytokines (TNF-*α*, IL-1*α*, IL-1*β*, IL-1RA, IL-6, IL-7, IL-10, IL-12p40, IL-12p70, IFN-*γ*, and TGF-*α*) and chemokines (IP-10, MIP-1*β*, MCP-1/CCL2, RANTES, and G-CSF) [59]. Similarly, VPA reduced the expression of CD80 and CD83 and limited the production of IL-6, IL-10, TNF-*α*, and IL-23 in LPS-activated moDCs. The altered production of IL-23 affected the ability of moDCs to promote the induction of Th17 cells [81].

**Figure 5:**
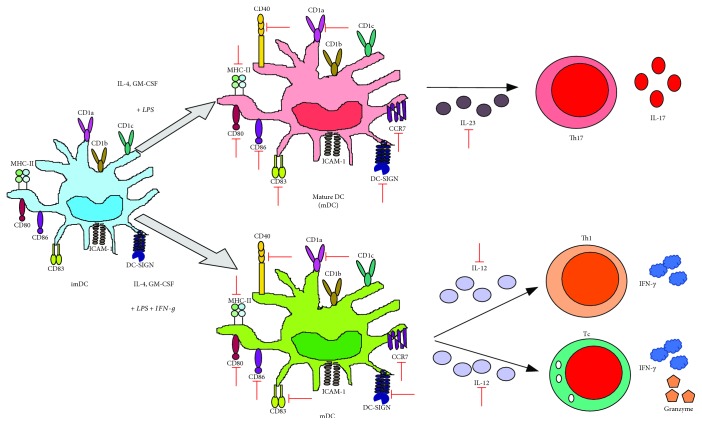
VPA impedes lymphocyte polarization by affecting the production of cytokines by mature DCs. VPA has diverse effects on DC maturation in response to either LPS or LPS+IFN-*γ*, including the reduction of costimulatory (CD40, CD80, CD83, and CD86), MHC-II (HLA-DR), adhesion (DC-SIGN), CD1a, and CCR7 (CCL19 receptor) molecules, which impedes chemotaxis of such cells, as well as IL-23 and IL-12 secretion, promoting Th17 or Th1 polarization, respectively. Green arrows indicate the processes, molecules, or mediators in the signaling pathway that are augmented and/or promoted by VPA. Red arrows indicate processes, molecules, or mediators in the signaling pathway that are inhibited by VPA.

Frikeche et al. found that VPA modified the phenotype of mature moDCs in response to LPS and IFN-*γ* by reducing the expression of CD83, CD86, and HLA-DR, as well as diminished the release of IL-10 and IL-12p70. This altered phenotype on moDCs relates with a failure to induce Th1 cells (CD4^+^, IFN-*γ*^+^) and a proper activation of CD8^+^ cytotoxic T cells (Tc), as evinced by the low production of IFN-*γ* and granzyme B [82]. The effect of VPA in the polarization of Th1, Th17, or Tc through the regulation of the cytokine environment by DC is outlined in Figure 5.

VPA also impairs the activation of pDCs by IL-3 and CpG by reducing the cell surface expression of CD40, CD80, CD83, ICOSL, PD-L1, and CCR7, while the release of IFN-*α*, IL-6, and TNF-*α* was diminished. VPA-exposed pDCs release significant amounts of IL-10 that interfere with the induction of Th1 cells [83].

### 3.4. Neutrophils

**Figure 6:**
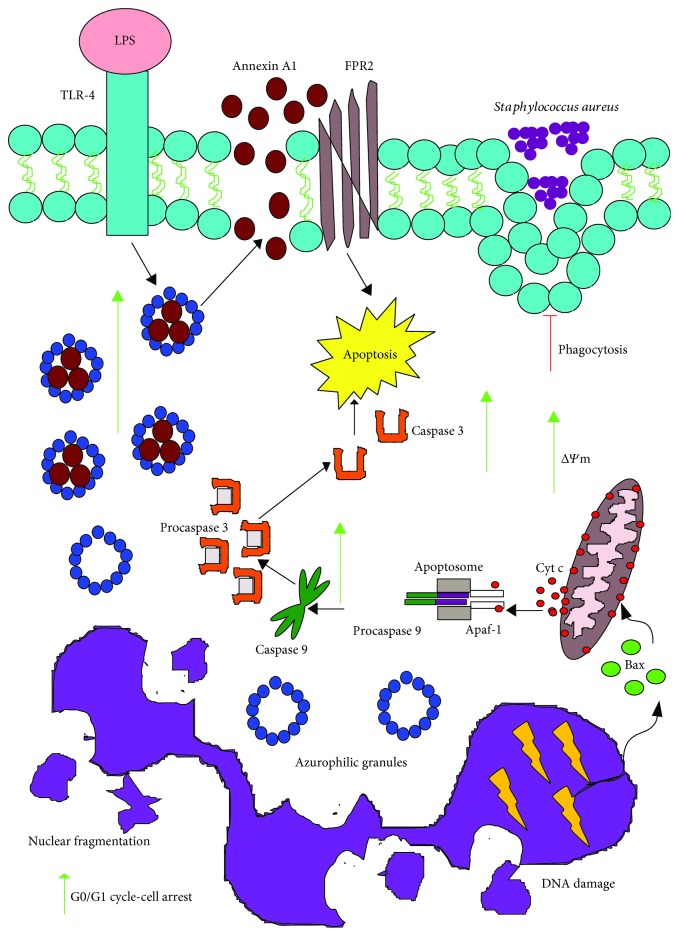
VPA induces apoptosis in neutrophils and diminishes their phagocytic capacity. VPA induces apoptosis in neutrophils in response to LPS, by increasing annexin A1 release. Additionally, VPA on its own can alter the mitochondrial membrane potential and increase activation of the both caspase 9 and caspase 3, which activate the apoptosis intrinsic pathway. Furthermore, VPA impedes proliferation by stopping the cells in the G0/G1 cell cycle phase. Finally, VPA reduces their phagocytic and antimicrobial activities against *Staphylococcus aureus*. Green arrows indicate the processes, molecules, or mediators in the signaling pathway that are augmented and/or promoted by VPA. Red arrows indicate processes, molecules, or mediators in the signaling pathway that are inhibited by VPA.

Bone marrow-derived neutrophils exposed to VPA and activated with LPS undergo apoptosis due to a rapid release of prestored annexin A1 in their granules (Figure 6) [68].

HL-60-differentiated neutrophils treated with VPA underwent cell cycle arrest during the G0/G1 phase and early apoptosis. This effect relates to the VPA-induced membrane potential reduction in the mitochondria, which is accompanied by the activation of caspase 3- and caspase 9-mediated apoptotic pathways (Figure 6) [84]. These observations explain the episodes of neutropenia observed in patients after prolonged therapy with VPA [85–87]. The reduction in neutrophil numbers was associated with the levels of circulating VPA [88, 89].

Neutrophils in patients chronically treated with VPA show an increased expression of the benzodiazepine receptor that lead to reduced chemotaxis to the N-formyl-methionine-leucyl-phenylalanine peptide, diminished phagocytosis, and elimination of *Staphylococcus aureus* (Figure 6) [90]. Neutrophils from epileptic infants chronically treated with VPA showed oxidative stress, as shown by the increased expression of myeloperoxidase (MPO) and malondialdehyde, a derivative of unsaturated fatty acid peroxidation. Moreover, neutrophils showed reduced levels of the antioxidant enzymes superoxide dismutase and catalase [91].

Mice that received VPA showed diminished neutrophil MPO activity in a model of acute lung injury [92] and reduced neutrophil infiltrates and proinflammatory cytokines in a model of spinal cord injury [72]. A VPA-induced reduction in neutrophil infiltrates in the bronchoalveolar fluid was also observed in a model of chronic lung inflammation due to cigarette smoke [89].

### 3.5. Eosinophils

The effect of VPA on eosinophil function has been poorly studied. However, patients developing eosinophilia in response to VPA treatment have been observed [93–97]. This effect is probably the result of a VPA-induced increase in systemic IL-5 levels [98].

### 3.6. Basophils

In mice that developed autoimmune lymphoproliferative syndrome (ALPS) due to Fas deficiency, treatment with VPA induced a reduction in the number of peripheral basophils, although the mechanism involved was not analyzed [99].

### 3.7. Mast Cells

In the HMC1.2 mast cell line, VPA diminishes proliferation and cell viability in a dose-dependent manner. Although the precise mechanism is not known, it is recognized that other HDACIs, such as SAHA, reduce the cell viability by inducing apoptosis and by reducing c-KIT expression [100].

### 3.8. NK Cells

VPA exhibits diverse effects on cellular development, including malignant cells from different cancers. VPA modulates the expression of stress molecules and other NKG2D ligands in tumor cells [46], which is used in the development of new therapies based on the use of NK cells as effector cells. However, whether VPA affects the activation mechanisms in NK cells has only recently been analyzed.

Some studies have observed that VPA reduces the cytolytic activity of human NK cells against diverse tumor cell lines. For instance, Pfeiffer et al. found that VPA reduces NK cytolysis of K562 and MHH-CALL-4 cells [101]. Similarly, VPA diminishes the lytic capacity of human NK lymphocytes in both leukemic (K562 and Jurkat) and hepatocarcinoma (HepG2) cells via an increase in the epigenetic signatures that repress the NKG2D receptor, which is essential in the lytic activity of NK cells. The epigenetic signatures included an increase in H3 Lysine 9 di-methylation (H3K9me2) near the *NKG2D* locus and at the promoter CpGs of this gene. Both changes correlated with downregulation in NK degranulation. VPA suppresses IFN-*γ* production in NK cells due to alterations in the phosphorylation of STAT-5. VPA-treated NK cells showed upregulation in PD-1 and PD-L1 expression, resulting in increased apoptosis [102].

VPA interferes with IL-2-dependent cytolytic activity of NK cells against tumor cells [103]. VPA diminishes the expression of the activator receptors NKp30 and NKp46, as well as the release of perforin and granzyme B, in response to IL-2. This effect was associated with reduced levels of activated NF-*κ*B and a blockade during the S-G2/M phase of the cell cycle. These alterations impaired NK cytolysis of the malignant cell lines K562, Jurkat, and HL-60 [104]. VPA reduces the cytotoxicity of NK cells in response to other cytokines including IL-12, IL-15, and IL-18, by downregulating NKG2D and NKp46 expression and inducing apoptosis, preventing NK cells from eradicating K562 tumor cells [105].

VPA reduced the cytolytic function of NK cells against malignant cells through the dysregulation of several receptors, mainly NKG2D. However, the underlying mechanism is not merely through methylation of the H3 histone at the *NKG2D* locus [106]. Accordingly, the expression of NKG2D depends on STAT3 phosphorylation and on low HDAC3-mediated acetylation levels at histones H3 and H4. VPA inhibition of HDAC3 alters STAT3 phosphorylation at tyrosine 705 and, together with the increased acetylation of H3 and H4 histones, leads to the suppression of NKG2D [107].

**Figure 7:**
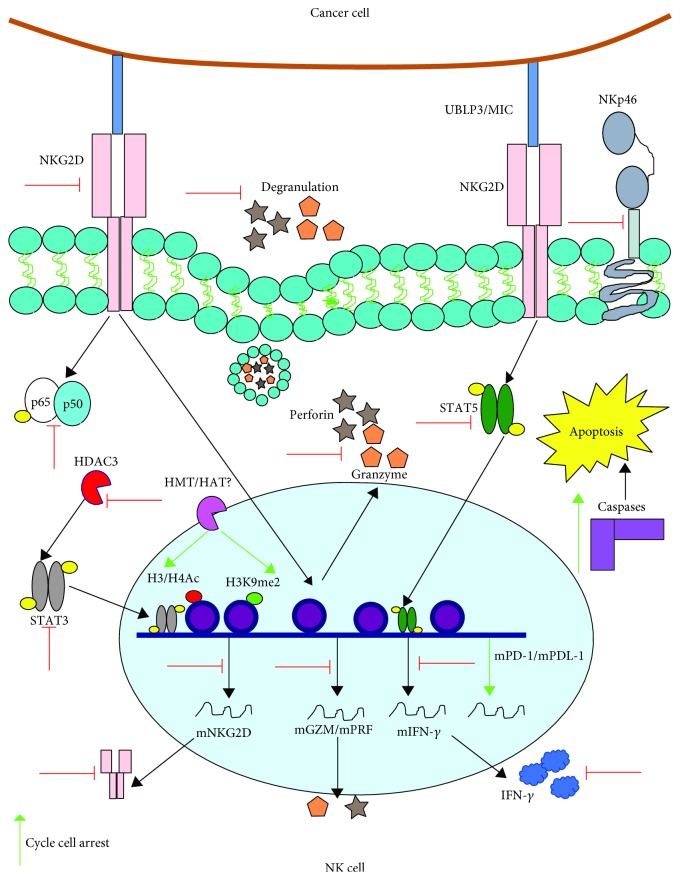
VPA reduces the cytolytic activity in NK lymphocytes against tumor cells. VPA reduces the expression of certain receptors that participate in the identification of tumor cells, including NKp46, NKp30 (not shown), and NKG2D. This last system of receptors recognizes overexpressed stress molecules (UBLP3/MIC) in malignant cells, which leads to the activation of different lytic-related activation pathways in the NK cell. It is feasible that VPA affects these pathways, including IFN-*γ* secretion by reducing STAT5 phosphorylation, as well as the production of both granzyme B and perforin, which are intimately linked to cellular degranulation. VPA also diminishes NF-*κ*B activation and the expression of NKG2D, by promoting the acetylation and methylation of the H3 histone that is next to the locus of this gene, and it blocks HDAC3, which strongly impacts STAT3 phosphorylation, which is required for the expression of this receptor. Furthermore, VPA blocks the cell cycle progression and increases the expression of both PD1 and the death ligand PD-L1, which is correlated with an increase in caspase expression and therefore with apoptosis. Green arrows indicate the processes, molecules, or mediators in the signaling pathway that are augmented and/or promoted by VPA. Red arrows indicate processes, molecules, or mediators in the signaling pathway that are inhibited by VPA.

These studies show that VPA reduces the cytolytic activity in NK cells by impairing various molecular effectors, hindering their capacity to eliminate tumor cells (Figure 7).

**Figure 8:**
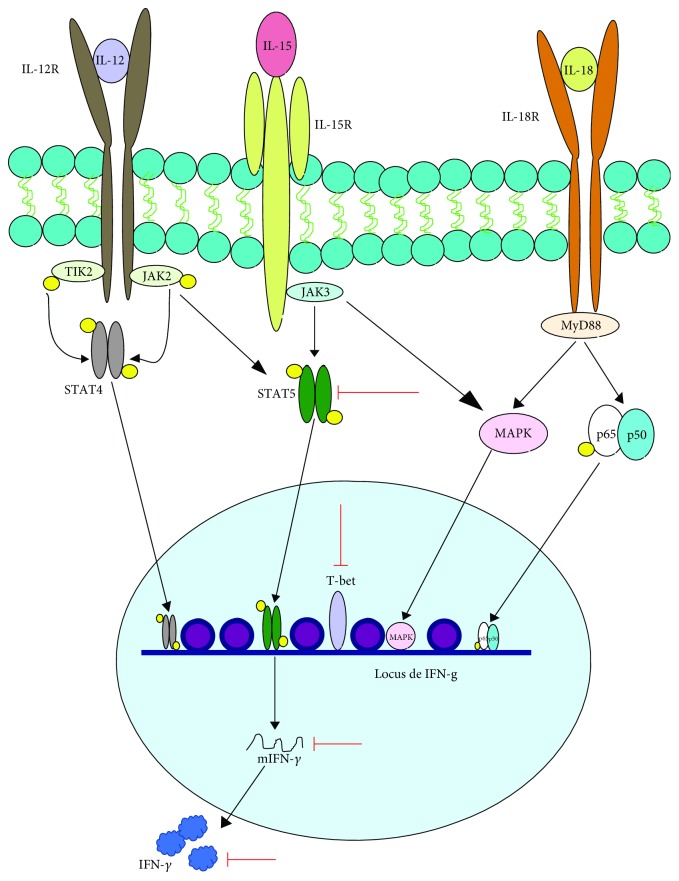
VPA affects IL-12-, IL-15-, and IL-18-mediated IFN-*γ* production in NK cells. NK cells produce IFN-*γ* through signaling pathways that are activated by the presence of IL-12, IL-15, and IL-18. In response to IL-12, STAT4 is activated and STAT5 is phosphorylated, while IL-15 activates both STAT5 and the MAPK pathway and IL-18 induces NF-*κ*B and the MAPK pathway. An intermediate of this path is p38 (not shown), which stabilizes the IFN-*γ* mRNA. This promotes a synergistic effect for the production of this cytokine. On the other hand, STAT5 activation promotes the expression of T-bet, recognized as the master regulator for IFN-*γ* expression. VPA also interferes with the production of this cytokine by reducing STAT5 phosphorylation and T-bet expression. Green arrows indicate the processes, molecules, or mediators in the signaling pathway that are augmented and/or promoted by VPA. Red arrows indicate processes, molecules, or mediators in the signaling pathway that are inhibited by VPA.

Mounting evidence shows that VPA also affects the production of IFN-*γ* in NK cells. Alvarez-Breckenridge et al. found that VPA reduced the production of IFN-*γ* in human NK cells after their exposure to IL-12, IL-15, and IL-18, via inhibition of phosphorylated STAT5 and the downregulation of T-bet. Furthermore, this effect correlated with the failed induction of cytotoxicity, due to the reduced expression of granzyme and perforin in glioblastoma cells infected with herpes virus simplex [108]. The adverse effect of VPA on IFN-*γ* production in response to IL-12, IL-15, and IL-18 was also observed in C57BL/6 mouse NK cells [105] (Figure 8).

VPA could be an alternative in the treatment of Epstein-Barr-related NK lymphoproliferative disorders because VPA limits the proliferation of NK cells, as observed in KAI3 and NKED cell lines infected with this virus. VPA favored the arrest at G1 of the cell cycle, which is associated with increased levels of p21^WAF1^, p27^KIP1^, and cyclin E, and a decrease in cyclin D2, CDK4, and C-Myc. VPA also induced apoptosis by upregulating caspase 3, caspase 8, and PARP and increasing the acetylation of H2B, H3, and H4 histones [109].

### 3.9. T*γδ* Cells

**Figure 9:**
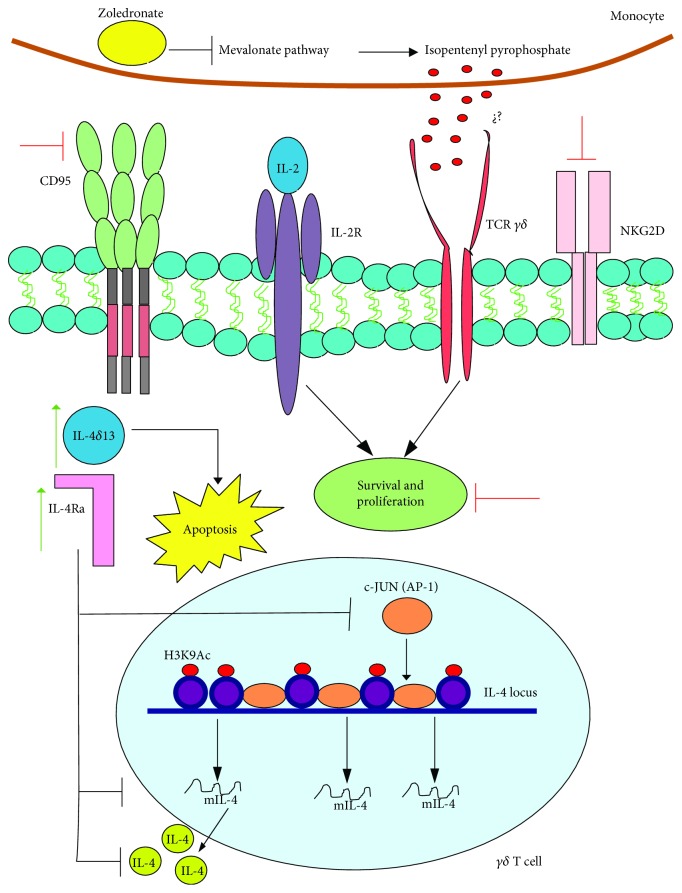
VPA induces apoptosis and inhibits IL-4 production in T*γδ* lymphocytes. The proliferation of T*γδ* lymphocytes arising from PBMC cultures, which are stimulated with IL-2 and zoledronic acid, is related to the promotion by the latter of the inhibition of the mevalonate pathway in monocytes, which upregulates pyrophosphate isopentenyl. This molecule is probably identified by the TCR of lymphocytes, but this has not been demonstrated. Furthermore, IL-2 sends proliferation and survival signals to these cells. However, VPA inhibits those mechanisms by promoting an increase in the intracellular molecules IL-4*δ*_13_ and IL-4R*α*, which have a direct effect on the induction of apoptosis and on the inhibition of IL-4 by blocking the AP1 signaling pathway. One additional VPA-related effect is the increase in H3 acetylation at the IL-4 locus, as well as a reduction in both CD95 and NKG2D. Green arrows indicate the processes, molecules, or mediators in the signaling pathway that are augmented and/or promoted by VPA. Red arrows indicate processes, molecules, or mediators in the signaling pathway that are inhibited by VPA. Figure based on [111].

VPA treatment inhibited the proliferation of human T*γδ* cells in response to zoledronic acid and IL-2. This inhibition was linked to an increase in apoptosis and H3 acetylation, while CD95 and NKG2D expression was reduced [110]. Later studies showed that VPA-mediated apoptosis was associated with increased production of IL-4*δ*_13_, which is an IL-4 variant related to apoptosis in T CD4^+^ lymphocytes infected with HIV (Figure 9) [111].

## 4. Effects of Valproic Acid on Cells of the Adaptive Immune Response

### 4.1. B Cells

**Figure 10:**
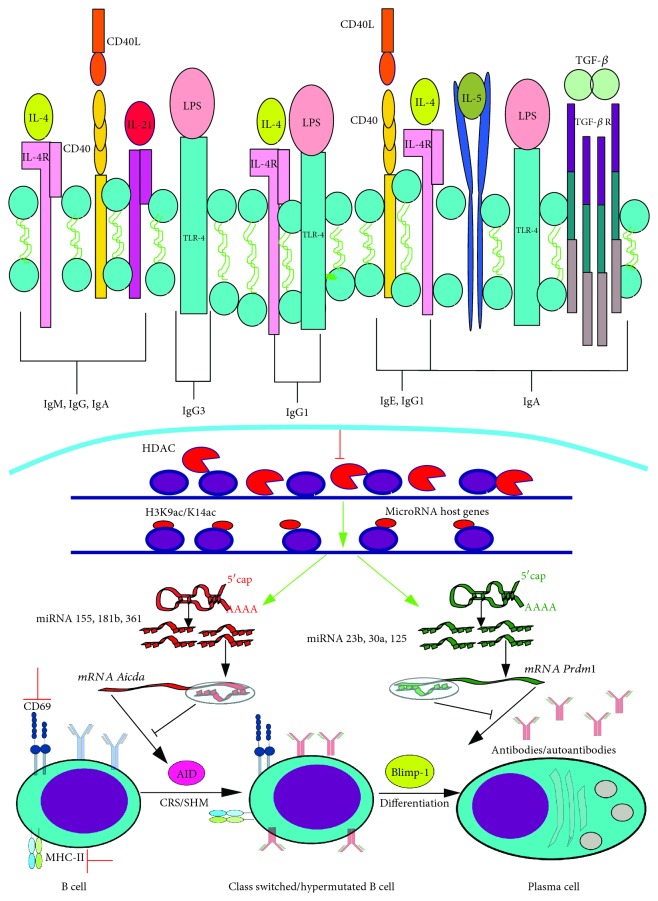
VPA impacts the B cell differentiation into plasma cells and in the isotype switch and maturation affinity of immunoglobulins. There are various signals by which B lymphocytes can differentiate into plasma cells that produce one or another immunoglobulin. CD40L+IL-21 promote IgM production and isotype switch into IgG and IgA; LPS+IL-4 induce isotype switch into IgG1; CD40L and IL-4 enable isotype switch into IgE; and IL-4+IL-5+LPS+TGF-*β*+dextran-conjugated anti IgD (not shown) prompt isotype switch into IgA. Isotype switch is mainly mediated by AID expression, while the naïve B lymphocyte differentiation into a plasma cell is governed by Blimp-1. VPA interferes with those mechanisms by inhibiting several HDACs, which promotes an increase in acetylation of the H3 that is next to the codifying genes for multiple miRNAs, which then block mRNA for AID (*Aicda*) and for Blimp-1 (*Prdm1*). Besides, VPA inhibits the expression of the early activation molecule CD69 and of MHC-II. Furthermore, this effect could be employed to reduce the generation of autoantibodies in mouse experimental lupus models. Green arrows indicate the processes, molecules, or mediators in the signaling pathway that are augmented and/or promoted by VPA. Red arrows indicate processes, molecules, or mediators in the signaling pathway that are inhibited by VPA. Figure based on [147].

Early studies demonstrated that VPA blocked the differentiation of naïve B cells in response to IL-21 and CD40L (Figure 10), as reflected by the reduction in CD27 and CD38 expression, which are plasma cell markers. Moreover, VPA reduced cellular activation by diminishing the expression of CD69 and MHC-II, affecting the production of IgM, IgG, and IgA without affecting CD40 and IL-21R levels [112].

*In vitro* and *in vivo* experiments showed that VPA controls B cell differentiation into plasma cells through silencing AID (an enzyme required for somatic hypermutation and class switch recombination) and Blimp-1 (an important transcription factor for differentiation into plasma cells). Such modifications reduce the number of plasma cells that produce IgG1, IgG2, IgG3, IgA, and IgE. VPA also affects the affinity of IgG1, IgG2, and IgG3 against thymus-dependent and thymus-independent antigens (Figure 10). This modulatory effect of VPA was used to reduce the autoantibody response in a mouse model of SLE, which contributed to increase mouse survival [113]. The mechanism involved in the silencing of AID and Blimp-1 by VPA was an increase in the expression of miRNAs 155, 181b, and 361, which target AID, while the increase of miRNAs 23b, 30a, and 125b was associated with the silencing of Blimp-1. These observations show that VPA can modulate gene expression in B cells through the expression of selected miRNAs [114].

Similar results were reported by Ye et al., who found that C57BL/6 mice treated with VPA one day before heart transplant from BALB/c mice showed diminished levels of specific IgG antibodies to the transplanted organ, and a reduction of plasma cells in the spleen. Those results suggest that VPA can be employed as a treatment to avoid antibody-mediated transplant rejection [115].

### 4.2. T Cytotoxic Lymphocytes

T CD8^+^ lymphocytes are affected by VPA, as shown by the reduction in cellular proliferation; modified expression of the activation markers CD69 and CD38; and diminished expression of HSP90 and FasL, which affects their cytolytic function [116].

Furthermore, T CD8^+^ lymphocytes from HIV-infected patients treated with VPA showed a decrease in the control of viral replication. Healthy donor-derived T CD8^+^ lymphocytes treated with VPA were unable to control viral replication in HIV-infected T CD4^+^ lymphocytes [117, 118]. This effect was related with the epigenetic suppression of noncytolytic mediators MIP-1*α*, lymphotoxin B, and IP-10 through an increase in H3 acetylation. This effect indicates that histone deacetylation, at least on T CD8^+^ lymphocytes, is crucial for the expression of noncytolytic mediators that promote the suppression of HIV replication. VPA also reduced the mRNA expression of other immunological components including IL-3, IL-22, CCR7, IL-8, and M-CSF [118]. Additionally, VPA showed toxicity in T CD8^+^ lymphocytes, limiting their ability to lyse human T-lymphotropic virus- (HTLV)- infected T CD4^+^ lymphocytes [119].

In experimental autoimmune encephalomyelitis (EAE) models, VPA reduced the percentage of T CD8^+^ lymphocytes in spleen and peripheral blood through caspase 3-mediated apoptosis. VPA similarly affected peripheral T CD8^+^ lymphocytes from donors and patients with multiple sclerosis [120]. This effect of VPA could be a potential alternative in the treatment of autoimmune diseases, in which the role of T CD8^+^ lymphocytes is essential. However, it should be noted that VPA has no effect on the viability or the activation of T CD8^+^ lymphocytes exposed to viral peptides [121]. This suggests that VPA may affect those functions depending on the immunological environment or the activation stimuli.

### 4.3. T Helper Lymphocytes (Th1, Th2, and Th17 Phenotypes)

**Figure 11:**
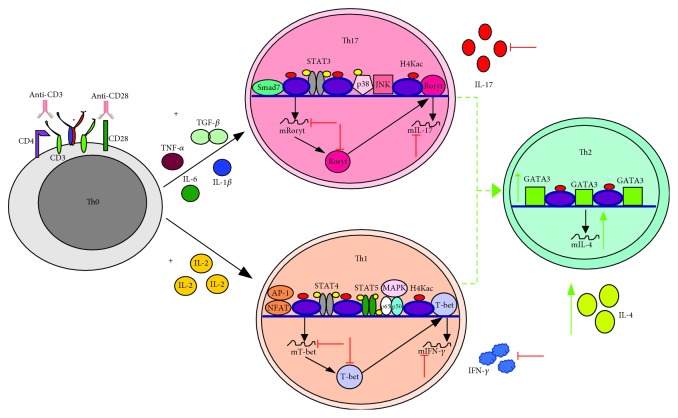
VPA polarizes Th1 and Th17 immune profiles into Th2 immune responses, both *in vitro* and *in vivo*. Th0, or naïve lymphocytes, that are activated *in vitro* with anti-CD3 and anti-CD28 and then polarized into Th1 with IL-2 or into Th17 with TGF-*β*+IL-6+TNF-*α*+IL-1*β* and which are then treated with VPA, are polarized toward a Th2 profile due to increased expression of GATA3 and IL-4. This reduces the expression levels of T-bet and IFN-*γ* (Th1), as well as of ROR*γ*t and IL-17 (Th17). This effect is also found in *in vivo* models of EAE, as well as in graft versus host disease. Additionally, VPA also induces apoptosis in Th1 and Th17, both *in vitro* and *in vivo* (not shown). Green arrows indicate the processes, molecules, or mediators in the signaling pathway that are augmented and/or promoted by VPA. Red arrows indicate processes, molecules, or mediators in the signaling pathway that are inhibited by VPA.

VPA promotes differentiation of naïve T lymphocytes toward a Th2 phenotype, increasing the expression of the transcriptional factor GATA-3 with a concomitant reduction in the expression of both T-bet and ROR*γ*t. VPA also diminishes the inflammatory processes related to Th1 and Th17 immune responses (Figure 11) [122]. Likewise, VPA reduces IFN-*γ* levels in T CD4^+^ lymphocytes stimulated with LPS [50] and increases the expression of NKG2D in Jurkat cells in response to IL-15 stimulation [123].

VPA blocks the production of IL-17A in Th17 lymphocytes [124]. On the other hand, naïve T CD4^+^ lymphocytes from mice, which are activated with anti-CD3/CD28 and polarized into either Th1 or Th17 phenotype, suffer apoptosis after exposure to VPA (Figure 11) [125].

In mouse models with EAE, VPA reduced T CD4^+^ lymphocyte infiltrates in the central nervous system, and it also diminished the percentage of Th1 and Th17 cells in the spleen and peripheral blood. This correlates with the induction of apoptosis mediated by caspase 3. The same effect was observed *ex vivo* in peripheral T CD4^+^ lymphocytes from both donors and multiple sclerosis patients [120]. In EAE rat models, VPA treatment reduced the percentage of peripheral CD4^+^ T lymphocytes and polarized the response toward a Th2 state by increasing the expression of GATA-3 and IL-4. This led to a decrease in both T-bet and IFN-*γ*, as well as in ROR*γ*t and IL-17 (Figure 11) [126], while in a rat model of experimental autoimmune neuritis, VPA reduced the number of Th17 lymphocytes in both the peripheral blood and sciatic nerve [127].

A similar effect occurred in graft versus host disease models. In irradiated BALB/c mice that received C57BL/6 mouse bone marrow cells and then were treated with VPA, the transplanted T CD4^+^ lymphocytes repopulated the spleen, liver, and lungs. However, these cells showed reduced intracellular levels of IFN-*γ* and IL-17A that correlated with low serum levels of these cytokines and reduced expression of T-bet and ROR*γ*t in T cells (Figure 11). The inhibition of the Th1 and Th17 responses was related to reduced levels of phosphorylated Akt and increased acetylation [128]. These results suggest that VPA is an exceptional agent for the control of highly exacerbated immune responses during autoimmune diseases.

VPA induces apoptosis of T CD4^+^ lymphocytes infected with HTLV, which impedes the increase in viral load due to increased expression of the viral proteins Tax, HBZ, and Gp19, suggesting a therapeutic use of VPA for HTLV-induced myelopathies [129]. Conversely, VPA upregulates the expression of the Tax gene in HTLV-infected T CD4^+^ lymphocytes [119], which makes lymphocytes excellent viral reservoirs that promote virus propagation.

In murine coxsackievirus-induced myocarditis models, VPA treatment diminished the percentage of Th17 lymphocytes and reduced IL-17A in serum. This effect was also observed ex vivo in T CD4^+^ lymphocytes infected with the virus in the presence of VPA [124].

In HIV-1-infected T CD4^+^ lymphocyte cell lines, VPA reduced the expression of the viral protein p24 during latency, while in LPS-stimulated peripheral lymphocytes, VPA reduced the expression of the HIV receptor [52]. This effect was also observed in the T cell line MT-2 cell, in which VPA reduced the expression of both CXCR4 and CD4, potentially inhibiting the reactivation of HIV-1 during latency [130]. This may explain why in some patients under conventional antiviral therapies, VPA reduces both the viremia and the percentage of infected CD4^+^ T cells [131, 132].

### 4.4. T Regulatory Lymphocytes

One of the most studied immunological effects of HDACIs, including VPA, is their ability to promote the generation of T regulatory lymphocytes (Treg) [122].

**Figure 12:**
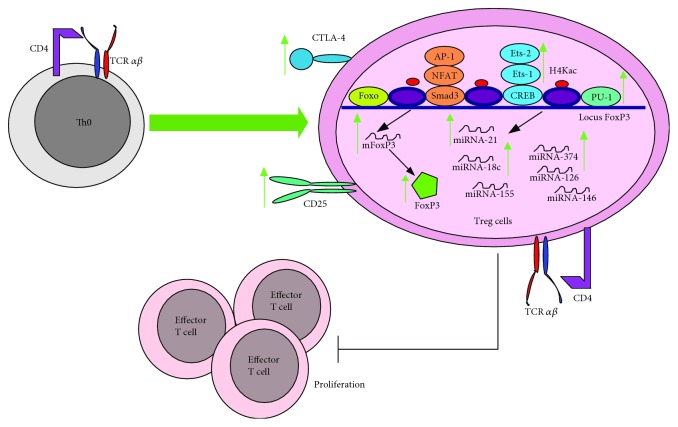
VPA promotes the generation of Treg cells. Naïve T lymphocytes that are treated with VPA differentiate into Treg cells due to epigenetic modifications (H4 histone acetylation) that allow the entry of the transcription factors Ets-1, Ets-2, and PU.1 into the promotor region of Foxp3, which induces its transcription and translation. VPA also increases the expression of miRNA-21, miRNA-18c, and miRNA-327, as well as of CD25, which are characteristic of Treg lymphocytes. However, it also induces the exhaustion molecule CTLA-4. Interestingly, VPA-induced Treg cells can block the proliferation of PBMC and T effector lymphocytes. Green arrows indicate the processes, molecules, or mediators in the signaling pathway that are augmented and/or promoted by VPA. Red arrows indicate processes, molecules, or mediators in the signaling pathway that are inhibited by VPA.

*In vitro* models showed that VPA induced the polarization of T CD4^+^/CD25^−^ lymphocytes into Treg cells by increasing the expression of Foxp3 via the acetylation of the histone H4 at the *FOXP3* promoter, allowing for the entry of the transcription factors Ets-1, Ets-2, and PU.1 to its promoter region. Moreover, VPA induced miRNA expression profile characteristic of natural Treg cells [133] and increased the half-life of Foxp3 [134]. In peripheral blood mononuclear cells (PBMCs), VPA induced Treg expansion after stimulation with anti-CD3 and anti-CD28. These cells expressed CD4^+^, CD25^+^, Foxp3^+^, and CTLA-4^+^ and inhibited T effector lymphocyte proliferation (Figure 12) [135].

On the other hand, naïve T CD4^+^ lymphocytes differentiated with TGF-*β*, anti-IFN-*γ*, and anti-IL-4 stimulation show an increased IL-10 production in the presence of VPA [124].

In the DBA/1 murine collagen-induced arthritis model, treatment with VPA expanded the Treg population that inhibited T effector proliferation [136]. A similar effect was observed in a mouse model of cystic fibrosis [137]. Furthermore, in a C57BL/6 DSS-induced colitis model, VPA increased the percentage of Treg cells in both the lymph nodes and spleen [125]. In a rat EAE model, the treatment with VPA increased mRNA expression of Foxp3^+^ and IL-10 in lymph nodes [126], and in rat experimental autoimmune neuritis models, VPA upregulated the number of Treg cells in both the peripheral blood and sciatic nerve [127].

In the murine coxsackievirus-induced myocarditis model, VPA treatment reduced viral loads and the expression of the capsid protein VP1 in the myocardium. However, it increased the percentage of Treg cells and IL-10 in serum. This effect was also observed *ex vivo*, where T CD4^+^ lymphocytes from the spleen of virus-infected mice cultured with VPA showed increased IL-10 production [124].

The ability of VPA to induce Treg is one mechanism that contributes to reduce the exacerbated Th1 and Th17 immune responses in autoimmune disease models. Altogether, current information suggests that VPA could be considered a very promising pharmacological candidate for the treatment of autoimmune diseases.

## 5. Final Remarks

Aside from its recognized actions in the field of neurology, VPA strongly impacts diverse activation mechanisms in both the innate and the adaptive components of the immune system. The evidence allows us to propose the use of this drug as a therapy against diverse pathologies, including autoimmunity, autoinflammation, and hypersensitivity.

Although there is an ongoing phase IV clinical trial using VPA in the control of bronchial asthma, results from the trial are still unavailable (NCT00153270). VPA could be an alternative for the control of an exacerbated immune response that occurs in tissue transplantation; this is because *in vitro* and *in vivo* findings indicate that VPA suppresses the inflammatory response mediated by cytokines, oxidative stress molecules (ROS, NO), activating receptors (NK, T*γδ*, and cytotoxic lymphocytes), perforin, granzyme, costimulatory molecules, and autoantibodies. Furthermore, VPA can polarize the immune response from Th1/M1 to Th2/M2, and it stimulates the generation of Treg cells.

The role of VPA on the induction of apoptosis induction could be employed in the control of ALPS, as observed in a clinical trial in which the administration of VPA reduced the size of the spleen and of the lymph nodes in patients with this disease (NCT00605657). However, the mechanisms by which VPA induces those effects are not clear.

Furthermore, VPA can be used in the treatment of certain infectious diseases. VPA could be effective against viral infections, because this drug not only affects the expression of the receptors and coreceptors used by viruses to initiate infection but also promotes apoptosis of infected cells. Similarly, though further studies are needed, VPA induces autophagy both in *in vitro* and in *in vivo* models, which can be an advantage against tuberculosis.

Although it was not within the scope of this review, the use of VPA in cancer treatment demands serious consideration, because several reports showed promising results of VPA against diverse forms of cancer. The evidence allows us to hypothesize that VPA might not only decrease tumor viability but also affect the immune cells. Therefore, a potential alternative could be the combination of VPA with cancer immunotherapy, as well as immune adjuvants including metabolic enhancers and/or growth factors and cytokines. This could be beneficial in the reduction of VPA dosages for the patient.

We expect new findings in the future regarding the complex mechanisms in the immune response elicited by VPA. Changes in the metabolism of immune cells depend on both the cell type and the stimuli from the microenvironment. Previous studies observed that immune cells with a proinflammatory phenotype (M1 macrophages, DCs, NK cells, Th1/Th2/Th17 lymphocytes, T CD8+ lymphocytes, and B lymphocytes) express one or more of the following metabolic pathways: glycolysis, pentose phosphate pathway, tricarboxylic acid cycle (TAC) truncated at two points (after citrate and after succinate), fatty acid synthesis, and catabolism of glutamine, arginine, and tryptophan. Immune cells with an anti-inflammatory phenotype (M2 macrophages, Treg cells) and memory T CD8+ lymphocytes expressed the TAC pathway, which is coupled with oxidative phosphorylation, as well as fatty acid *β*-oxidation. In M2 macrophages the catabolism of glutamine and arginine is prevalent [138, 139].

Innate immune cells, including monocytes, macrophages, and NK lymphocytes, which are activated by PAMPs, can respond better over a second encounter with the same microbial or other nonrelated stimuli. This process, which is known as innate immune memory or immune training, is related to metabolic changes such as an increase in glucose uptake, with anaerobic glycolysis secondary to the mTOR-HIF1*α* activation and with a reduction in TCA activity and oxidative phosphorylation. Other induced metabolic pathways are the pentose phosphate pathway, fatty acid and cholesterol biosynthesis, and accumulation of TCA intermediates (citrate, succinate, malate, and fumarate), probably replenished by glutaminolysis. Some of these molecules work like substrates for different epigenetic enzymes. As such, NAD+ is a substrate for HDAC and acetyl-CoA for HAT, while both fumarate and succinate inhibit the activity of lysine demethylases; this imprints a characteristic epigenetic pattern in training cells: H3K9ac, H3K27ac, H4Kac, H3Kme1, H3Kme3, and loss of DNA methylations. The final result is the augmented expression of proinflammatory cytokines and PRRs [140–142].

Some information exists about the VPA-induced epigenetic marks on monocytes (H3Kac and HK4ac) [43], splenocytes [45], and H3Kac, H4KAc [104], and H3Kme2, as well as CpG DNA methylation on NK cells. However, there is no information showing the effect of VPA in the metabolism of immune cells. Multiple studies showed that VPA affects certain metabolic pathways in other cells. In neurons, VPA impairs some enzymes of the Krebs cycle [1]; in hepatocytes, it promotes the accumulation of both the lactate and pyruvate [143, 144]. This could be related to a higher glycolytic rate or to the inhibition of pyruvate uptake by mitochondria. Therefore, coenzyme-A-dependent TCA enzymes are inhibited [145], and oxidative phosphorylation is blocked as well [146]. Such effects on hepatocyte metabolism are similar to those observed in the immune training, which leads to the next question: could VPA work as an inducer of immune training?

## Figures and Tables

**Table 1:** Employment of valproic acid in clinical trials.

Group of diseases	Specific condition in which VPA has been employed	ClinicalTrials.gov identifier:
Neurological disorders	Epilepsy	NCT00385411
	Alzheimer's disease	NCT01729598
	Neuropathic pain	NCT00221637
	Amyotrophic lateral sclerosis	NCT00136110
	Delirium	NCT02343575
	Autism	NCT00211796
	Psychotic disorder	NCT01094249
	Bipolar disorder	NCT00431522
	Depression	NCT00186186
	Migraine	NCT00195741
	Dementia	NCT00315900
	Schizophrenia	NCT00194025
	Spinal muscular atrophy	NCT00661453
	Neuralgia	NCT00221637
	Posttraumatic stress	NCT00203385
	Attention deficit hyperactivity disorder	NCT00228046

Dependencies	Alcohol abuse	NCT01342549
	Cocaine dependence	NCT00240110
	Marijuana abuse	NCT00218114
	Opiate dependence	NCT00367874

Cancer	Glioma	NCT00302159
	Astrocytoma	NCT03243461
	Melanoma	NCT00358319
	Colorectal cancer	NCT01898104
	Malignant mesothelioma	NCT00634205
	Lymphoma	NCT00854581
	Chronic lymphocytic leukemia	NCT02144623
	Acute myeloid leukemia	NCT00414310
	Thyroid neoplasia	NCT01182285
	Brain metastasis	NCT00513162
	Breast cancer	NCT01010854
	Nasopharyngeal cancer	NCT00181220
	Prostate cancer	NCT00670046
	Bladder cancer	NCT01738815
	Kaposi's sarcoma	NCT00075777
	Small cell lung carcinoma	NCT00759824

Others	Bronchial asthma	NCT00153270
	Autoimmune lymphoproliferative syndrome	NCT00605657
	Hypersplenism and lymphadenopathy	NCT00605657
	Retinitis pigmentosa	NCT01233609
	Focal glomerulosclerosis	NCT02896270
	Insulin resistance	NCT00552500
	Androgenic alopecia	NCT01548066
	Hematuria	NCT01738815
	HIV viral replication control	NCT00289952

**Table 2:** Effects of valproic acid on some cellular elements of the immune response.

Cell	Observed effects	References
Monocytes	Reduction of proinflammatory cytokines	[49, 50]
	Reduction of surface molecules	[52]

Macrophages	Reduction of proinflammatory cytokines	[56, 58–61, 66, 76]
	Reduction of costimulatory molecules	[61]
	Reduction of ROS and NO	[57, 58, 63]
	Reduction of phagocytosis of extracellular pathogens	[63]
	*Mycobacterium tuberculosis* replication control	[64–67]

Dendritic cells	Reduction of proinflammatory cytokines	[59, 81, 82]
	Reduction of costimulatory molecules	[59, 79–82]
	Reduction of MHC-II	[82]

Neutrophils	Reduction of phagocytosis	[90]
	Reduction of chemotaxis	[90]
	Cellular arrest	[84]
	Apoptosis	[68]

Eosinophils	Eosinophilia in some clinical studies	[93–97]

Basophils	Reduction in total numbers in mouse autoimmune lymphoproliferative syndrome models	[99]

Mast cells	Reduction of cellular proliferation and viability	[100]

NK lymphocytes	Reduction of cellular proliferation	[109]
	Reduction of cytotoxic activity	[101–106]

T*γδ* lymphocytes	Apoptosis	[110, 111]

B cells	Inhibition of differentiation of B cell into plasma cell	[112–115]

T CD4^+^ lymphocytes	Reduction of cellular viability	[120, 129]
	Reduction in proinflammatory cytokines	[50, 124, 126, 128]

T CD8^+^ lymphocytes	Reduction of cytotoxic activity	[116]
	Reduction of cellular viability	[120]

Treg lymphocytes	Increase of cellular proliferation	[122, 124, 125, 127, 135, 136]
	Increase in FoxP3 expression	[126, 133, 134]
	Increase of suppressor activity	[135–137]

ROS: reactive oxygen species; NO: nitric oxide; MHC-II: major histocompatibility molecule II.

## Abbreviations

VPA: Valproic acid
HDACI: Histone deacetylase inhibitor
CYP450: Cytochrome P450
GABA: *γ*-Aminobutyric acid
GAD: Glutamine acid decarboxylase
SSAD: Succinic semialdehyde dehydrogenase
GABA-T: GABA transaminase
PET/CT: Positron emission tomography/computed tomography
HSPC: Hematopoietic stem/progenitor cells
NO: Nitric oxide
iNOS: Inducible nitric oxide synthase
BMDM: Bone marrow-derived macrophages
ROS: Reactive oxygen species
PARP3: Poly(ADP-ribose) polymerase 3
AAM: Alternatively activated macrophage
SLE: Systemic lupus erythematous
DC: Dendritic cells
pDC: Plasmacytoid dendritic cells
moDC: Monocyte differentiation into dendritic cells
GM-CSF: Granulocyte-macrophage colony-stimulating factor
MPO: Myeloperoxidase
WBC: White blood cell
ALPS: Autoimmune lymphoproliferative syndrome
HTLV: Human T-lymphotropic virus
EAE: Experimental autoimmune encephalomyelitis
Treg: T regulatory lymphocytes
PMBC: Peripheral blood mononuclear cells
TAC: Tricarboxylic acid cycle.
